# A chronic EBV infection causing persistent facial erythema multiforme and a retrospective literature review: A case report

**DOI:** 10.1097/MD.0000000000031865

**Published:** 2022-12-23

**Authors:** Peng Fenfang, Guo Hui

**Affiliations:** a Department of Pediatrics, West China Second University Hospital, Sichuan University, Chengdu, Sichuan, China; b Key Laboratory of Birth Defects and Related Diseases in Women and Children, Sichuan University, Ministry of Education, Chengdu, Sichuan, China.

**Keywords:** chronic EBV infection, erythema multiforme, facial lesion, Henoch-Schonlein purpura, systemic lupus erythematosus

## Abstract

Epstein-Barr virus (EBV) infection is associated with a variety of diseases and can involve multiple organs and systems, with complex and nonspecific clinical manifestations that are easily misdiagnosed. Chronic EBV infection with persistent erythema multiforme (EM) on the cheek as the main manifestation is very rare and has been reported rarely.

**Patient Concerns:**

This article reports a case of an adolescent female with chronic EBV infection who presented with chronic symmetrical erythema lesions on the face for 4 years, exacerbated with photophobia, lacrimation, Henoch-Schonlein purpura (HSP)-like rash, decline in granulocyte and erythrocyte lineages, hematuria, and proteinuria for 1 week.

**Diagnoses:**

The disease was initially misdiagnosed as systemic lupus erythematosus (SLE) and later confirmed as chronic EBV infection by skin biopsy. In the case, EBV infection not only caused chronic facial EM, but also induced acute HSP and purpura nephritis (hematuria and proteinuria type).

**Interventions:**

The child was treated with 1 week of glucocorticosteroids in adequate doses combined with acyclovir antiviral therapy and 3 sessions of hemoperfusion. After discharge, she took prednisone acetate (15 mg twice a day) orally for 1 month and then discontinued.

**Outcomes:**

She was discharged with her rash relieved and normal blood routine test and urine routine test. After 13 months of long-term follow-up, her facial erythema and hyperpigmentation became lighter, and there was no new rash on the whole body, and no abnormality in continuous monitoring of complete blood count and urine test.

**Lessons:**

This case suggests the need to be alert for chronic EBV infection in adolescent females with chronic facial EM rash and multiple organs and systems injury, in addition to connective tissue diseases such as SLE.

## 1. Introduction

EBV was discovered in 1964 by Epstein and Barr et al in lymphoma patients^[1]^ and belongs to γ-herpesvirus, a double-stranded (ds) deoxyribonucleic acid (DNA) virus that infects B lymphocytes^[2]^ and to which the population is generally susceptible.^[3]^ EBV is spread via saliva, leading to infection of oral epithelial cells, followed by infection of B lymphocytes. After 4 to 6 weeks of incubation, the virus replicates in large numbers in B cells.^[4]^ These B cells migrate to the lymph nodes and peripheral blood to induce cellular and humoral immunity. The body relies primarily on CD8 + T cells for recognition and clearance of EBV, but they cannot be eliminated, and very few are latent within B cells to form latent infections that remain with the host for life. Most EBV infections are insidious or mild onset, while a few may show persistent viral activity or recurrent activation after virus latency.

EBV infection can induce a variety of diseases. It is the pathogen of infectious mononucleosis (IM) and is also closely associated with the development of nasopharyngeal carcinoma and childhood lymphoma, and is classified as one of the human oncogenic viruses that may cause cancer. EBV infection is not only associated with a variety of diseases, but also has a complex and diverse clinical presentation that can involve multiple organs and systems and is nonspecific to be easily misdiagnosed. EBV infection is associated with a variety of skin diseases, including SLE, dermatomyositis, scleroderma, EM, HSP, urticaria, and atopic dermatitis.

EBV belongs to human herpesvirus IV, and the population is generally susceptible. Primary EBV infections are common in children and tend to be asymptomatic or present as IM. After primary EBV infection, most patients are in latent infection state, while some patients present with chronic active EBV infection. In recent years, EBV infection has been found to be associated with several skin diseases, such as drug induced hypersensitivity syndrome, hydroa vacciniforme (HV), and extranodal natural killer/T cell lymphoma (nasal type). The skin lesions of EBV infection are diverse and nonspecific, including EM, pityriasis lichenoides et varioliformis acuta, facial inflammatory granuloma annulare etc.^[1,5–6]^ Most cases of EM occur after IM symptoms of EBV infection, a few have no prior IM symptoms, and the erythema is often self-limiting. Persistent EM is a rare clinical subtype of EM.^[7]^ Chronic active EBV infection is also a rare outcome of EBV infection.^[8]^ This article reports a case of chronic active EBV infection in a child presenting with persistent facial EM.

## 2. Ethics approval

Written informed consent was obtained from the parents of the patient for her anonymized clinical and genetic data to be analyzed and published for research purposes. This study was approved by the Ethics Committee of the West China Second Hospital of Sichuan University, Chengdu, China.

## 3. Case report

**Figure 1.**
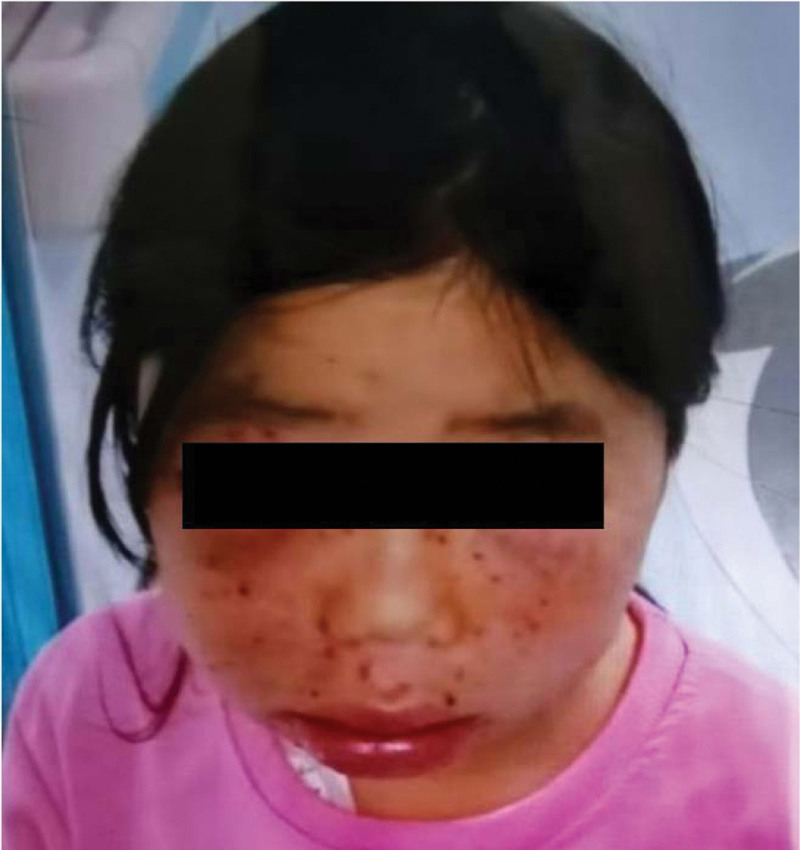
Bilateral symmetric erythema with scattered purplish papules on the cheeks 4 years ago.

**Figure 2.**
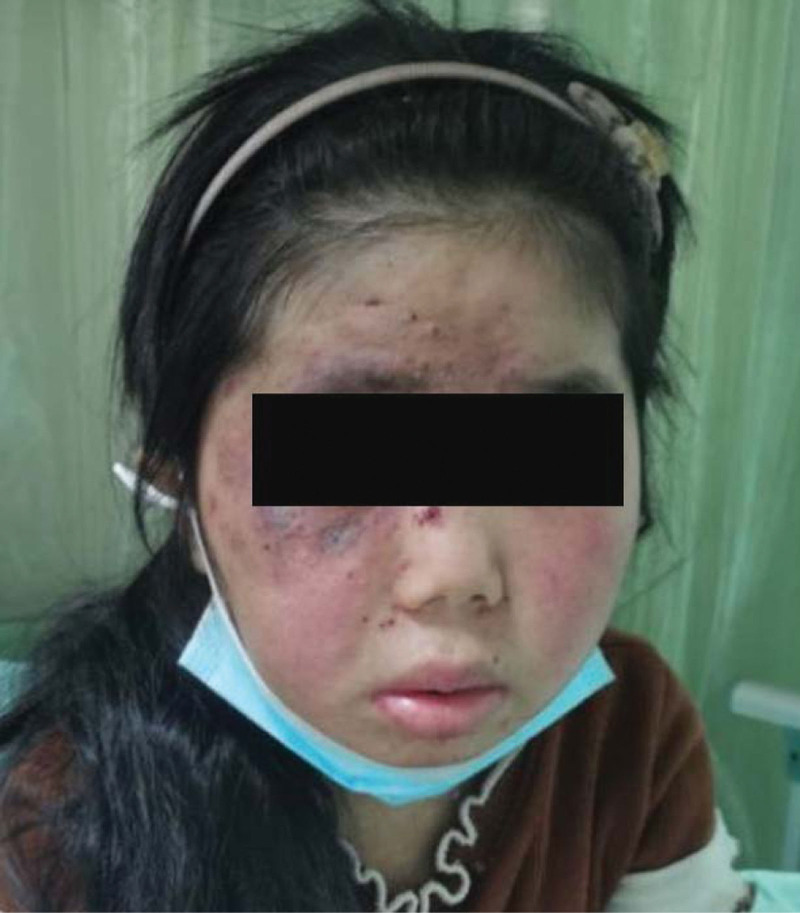
Bilateral symmetric erythema on the cheeks, with target-shaped lesion with hyperpigmentation visible on the right.

**Figure 3.**
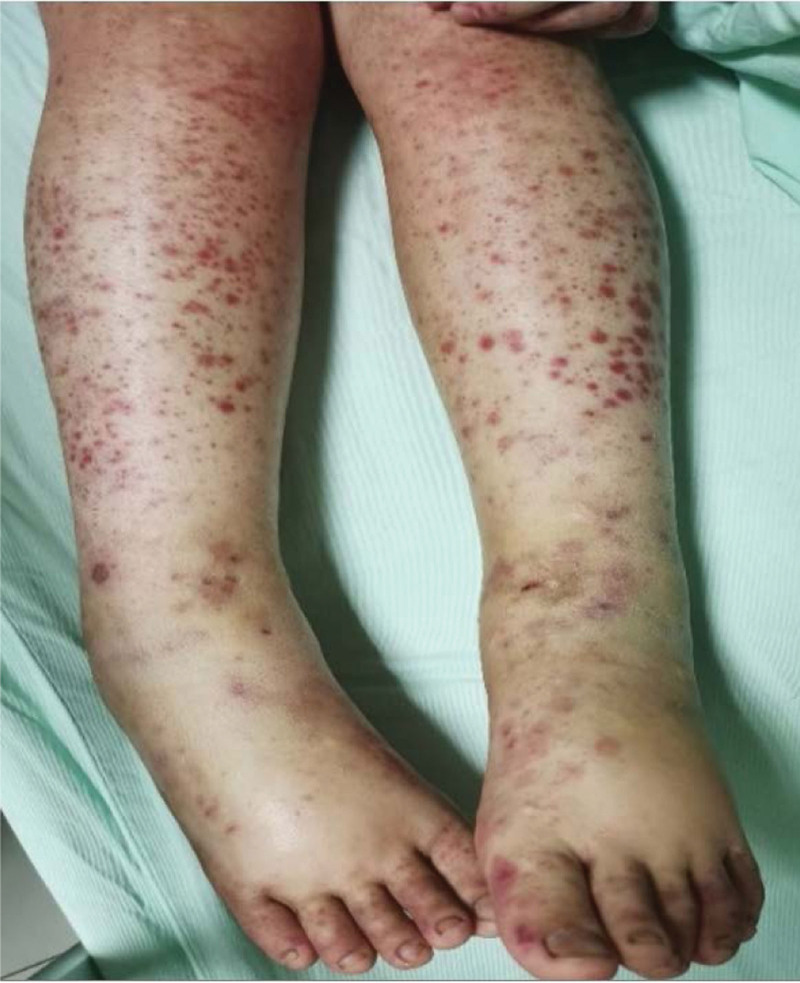
Symmetrical distribution of purple-red macules with scattered large herpes on both lower limbs, some of which rupture and crust.

**Figure 4.**
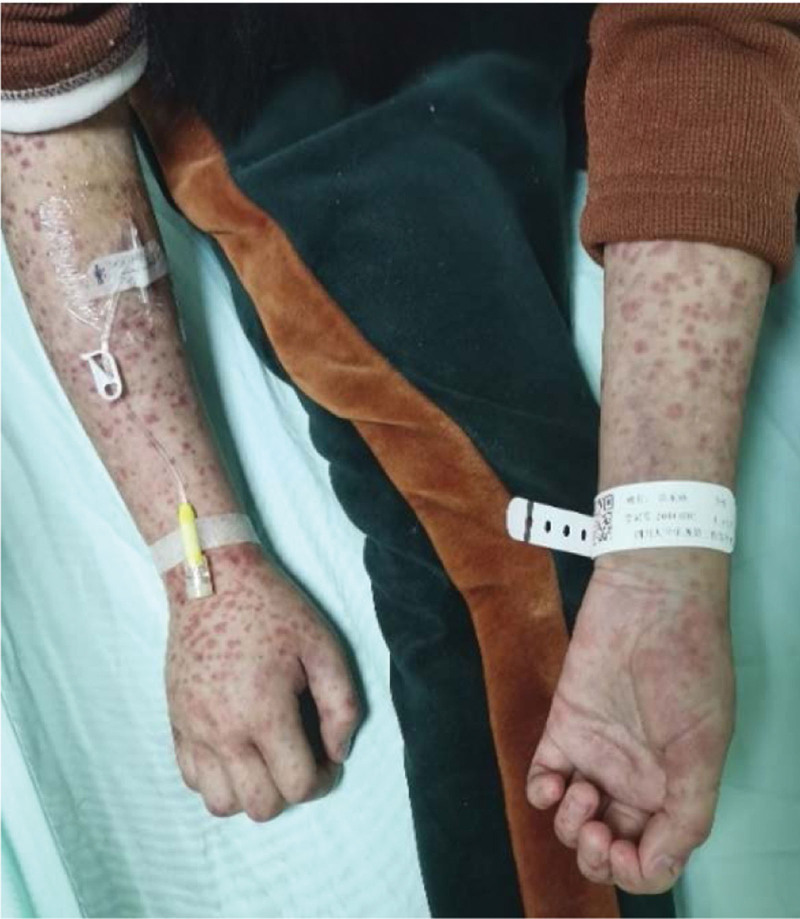
Symmetrical distribution of purplish red macules on both upper limbs.

A female aged 11 years and 8 months was admitted to the hospital for facial rash for 4 years, exacerbated with abdominal pain and arthralgia for 1 week. Four years ago, the child developed symmetrical erythema with scattered purplish papules on the cheeks bilaterally without any obvious cause (Fig. 1). Afterwards, the child’s facial rash spread slowly and was treated with prednisone acetate (20 mg twice a day) orally for 3 months without improvement, and then was not treated. One week before admission, the child developed a diffuse symmetrical distribution of purple-red maculopapular, mainly on the limbs, above the skin surface, which did not fade when pressed and partially fused into patches. Scattered bullous eruptions were also seen partially broken and crusted without desquamation (Fig. 2–4). In addition, the child had abdominal pain, arthralgia, photophobia and lacrimation, and was admitted for further treatment. After admission, complete blood count showed decreased leukocytes (2.3 × 10^9^/L), neutrophils (1.33 × 10^9^/L) and hemoglobin (94 g/L); repeated urine tests showed the presence of proteinuria and glomerular-derived hematuria (protein+, erythrocytes 4+/HP, fine granular tubularity detected). Diagnosis:

1. purpura nephritis (hematuria and proteinuria type).
2. HSP.
3. SLE.

**Figure 5.**
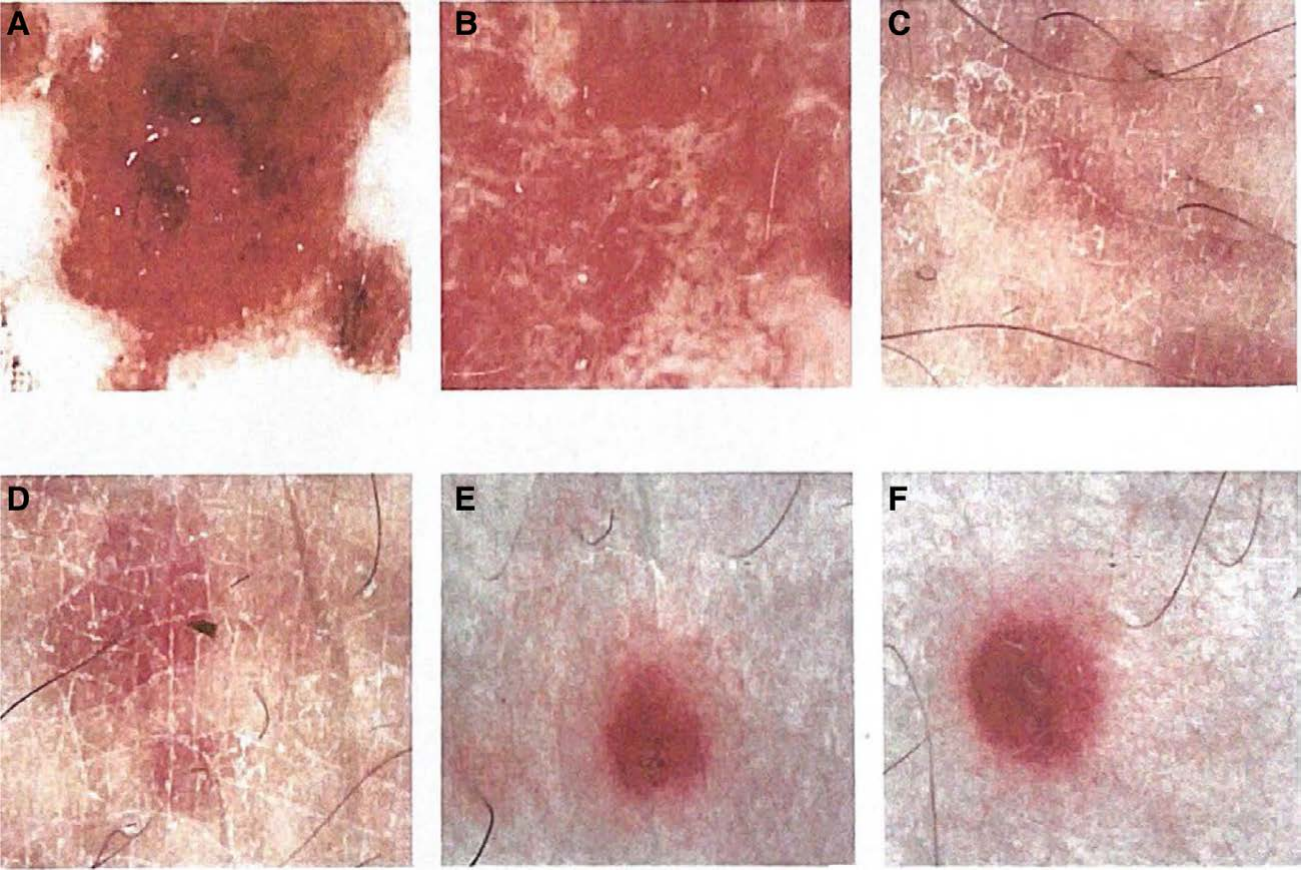
Dermatoscopy report. (A) Face (middle), polarized light. Scattered few white scales are seen on a red background, with visible blood crusts, dotted bulbous vessels and hemorrhagic spots on the margins, and no obvious pigmented structures are seen. (B) Face (right), polarized light. Many thicker linear branching vessels are seen on a pink ground, with few visible white scales, many regularly distributed white structureless areas, and no obvious pigmented structures are seen. (C, D) Upper limb (right), polarized light. (E, F) Lower limb (right), polarized light. Multiple purplish-red erythema with uneven color and well-defined borders are seen, and linear branched vessels can be seen in some lesions.

**Figure 6.**
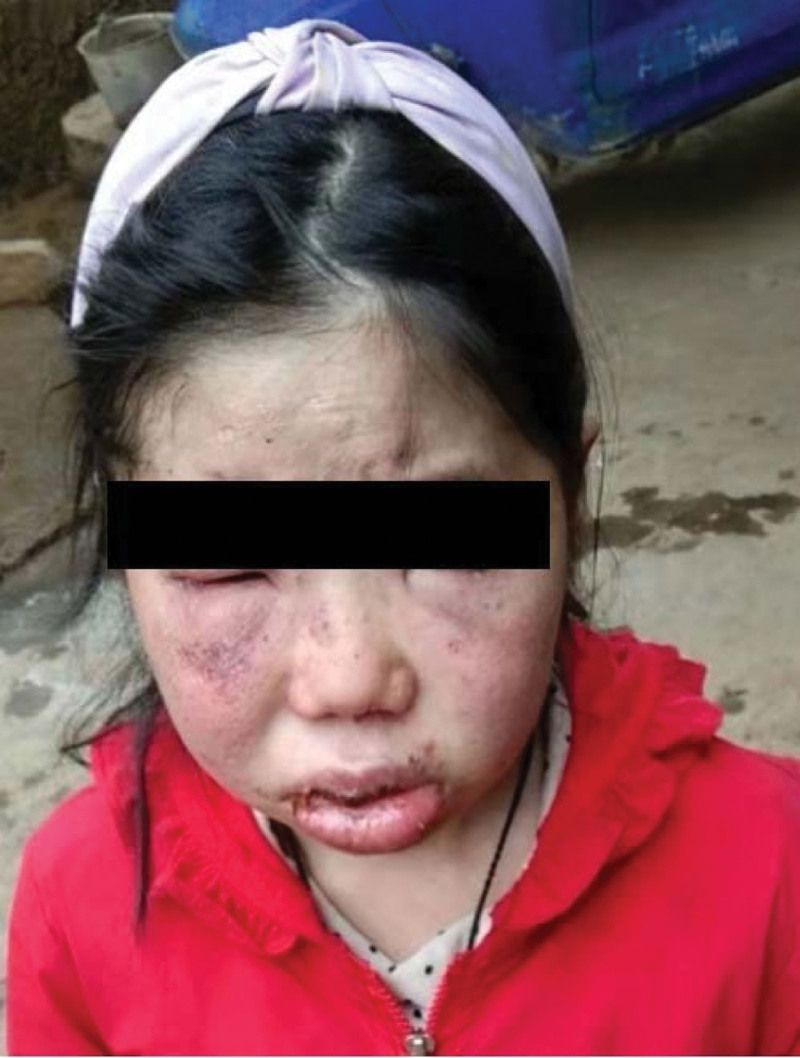
Facial erythema and hyperpigmentation faded and no rash.

After 1 week of anti-inflammatory treatment with intravenous methylprednisolone (32 mg once a day), the child’s rash, abdominal pain and arthralgia did not improve significantly. Further tests of autoantibodies, ANCA, humoral immunity, cellular immunity, tumor markers, bone marrow smear, herpes simplex virus, mycoplasma, chlamydia, fungal G, and tuberculosis antibodies showed no abnormalities. Anti-EBV early antigen IgG (EA-IgG), anti-EBV capsid antigen IgG (viral capsid antigen [VCA]-IgG) and anti-EBV nuclear antigen IgG were positive, while anti-EBV capsid antigen IgM was negative and anti-EBV capsid antigen IgG had high affinity. EBV viral load was 2.23 × 10^3^ copies/mL. The possibility of skin and kidney injury due to EBV infection was considered high, so further dermatopathological tests were performed. Dermatoscopy report of face, right upper and lower limbs (Fig. 5): under polarized light, multiple purplish-red erythema with uneven color and well-defined borders were seen, and linear branched vessels could be seen in some lesions. Dermatopathology report of the right face: the epidermis was approximately normal, while the entire dermis and the superficial layer of subcutaneous fat were infiltrated by many dense lymphocytes, most of which were medium and were irregular in nuclear morphology. Dermatopathology report of the nasal bridge: the whole dermis and the superficial layer of subcutaneous fat were infiltrated by many dense equally medium lymphocytes with irregular nuclear morphology, and individual eosinophil infiltration was seen. Dermatopathology report of the left calf: the epidermis was approximately normal, while the dermis was infiltrated in restricted areas with moderate amounts of medium lymphocytes, some neutrophils and nuclear fragmentation, and extensive erythrocyte exudation was among them. Immunohistochemical tests on the skin of right face and nasal bridge were consistent with the manifestation of chronic active EBV infection as a cutaneous lymphoproliferative disorder. After 1 week of treatment with glucocorticosteroids in adequate doses combined with acyclovir antiviral therapy and 3 sessions of hemoperfusion, the rash on the face, limbs and trunk of the child subsided, abdominal pain and arthralgia subsided, and complete blood count returned to normal. The child was discharged with oral prednisone acetate (15 mg twice a day) for 1 month and then discontinued. After 13 months of long-term follow-up, the child’s facial erythema and hyperpigmentation became lighter (Fig. 6), and there was no new rash on the whole body, and no abnormality in continuous monitoring of complete blood count and urine test.

## 4. Discussion

In this case, the child had chronic exacerbation of symmetrical EM on the cheeks because of persistent viral activity after EBV infection. EM is an acute, self-limiting autoimmune disease characterized by a typical target-like rash,^[9]^ which predominates in young men and is rare in children.^[10]^ Persistent EM usually lasts for months to years and may produce a more severe rash than regular EM.^[11]^ This case is a near-adolescent girl with persistent erythema on the cheek for 4+ years. One week before admission, a more inflammatory bullous rash with residual hyperpigmentation than normal EM appeared, which is consistent with the characteristics of persistent EM. Persistent EM is a rare clinical subtype of EM^[7]^ and is associated with EBV infection, herpesvirus infection, malignancy, inflammatory bowel disease, and herpes simplex virus infection.^[12]^ The etiology of persistent EM is predominantly herpesvirus infection, and EBV infection is extremely rare.^[11]^ In this case, persistent EM was confirmed by skin biopsy to be associated with chronic active EBV infection. This suggests that adolescent female children presenting with symmetrical facial erythema with multiple organs and systems injury should be alerted to CAEBV infection.

In this case, EBV infection caused not only persistent EM but also HSP. There are not many reported cases of HSP induced by EBV infection. The incidence of HSP induced by EBV infection was 4.2%, while the rate of EBV infection in children with HSP was 0.9%.^[13]^ EBV can be latent in human lymphoid tissues for a long time, and when the body’s immunity decreases, it proliferates and destroys B lymphocytes, leading to a change in B lymphocytes surface antigens, which then triggers a T lymphocytes defense response and causes a large secretion of inflammatory mediators. Positive IgG antibodies to the capsid antigen, positive IgG antibodies to the nuclear antigen and high affinity of IgG antibodies to the capsid antigen in the child suggest previous infection. Considering that the skin immunohistochemical findings at the polymorphic erythema of the face and nasal bridge were consistent with the manifestation of CAEBV infection, and that the EBV viral load was significantly elevated on this admission (2.23 × 10^3^ copies/mL), this suggested that the child had further activation of EBV on top of CAEBV infection. Meanwhile, the child presented with a diffuse generalized hemorrhagic purplish papule with abdominal pain and arthralgia, suggesting that HSP was induced by EBV reactivation or further activation. HSP is an IgA-mediated vasculitis, and infection is the main cause of its development.^[14,15]^ The continuous depletion of plasma IgA caused by HSP leads to massive proliferation of EBV, which can stimulate the body to produce more immune complexes, resulting in damage to the glomerular basement and filtration membranes or directly exerting viral cytopathic effects (immune complex injury or direct viral cytopathic effect is one of the potential mechanisms), thus further exacerbates the HSP condition.^[16]^ EBV infection is a risk factor for secondary renal damage in HSP and one of the factors that make HSP difficult to treat EBV.^[15]^ One week before admission, this child presented with a large purpura-like rash with rupture and necrosis, combined with renal damage (hematuria and proteinuria), suggesting severe vasculitis due to active EBV. After the first anti-inflammatory treatment with methylprednisolone, the rash was not resolved as expected, but the symptoms improved significantly after the combination of acyclovir antiviral and 3 sessions of hemoperfusion to remove immune complexes from the plasma, which suggested the importance of anti-EBV treatment.

In this case, a near-adolescent female with persistent viral activity after EBV infection developed chronic exacerbation of symmetrical EM on the cheek, which is similar to that of SLE causing facial butterfly erythema. Meantime, the child had multiple organs and systems injury such as photophobia, lacrimation, HSP-like rash, decline in granulocyte and megakaryocyte lineages, hematuria and proteinuria, which were easily misdiagnosed as SLE. Numerous studies have shown that EBV infection is strongly associated with SLE. Neelakshi R et al described the mechanisms of immune dysregulation due to EBV and the involvement of these mechanisms in the development and progression of SLE.^[17]^ Quaglia M et al confirmed that EBV will induce or exacerbate SLE in genetically susceptible patients and explained that EBV-specific protein regulation interacts with the host immune system to promote SLE through immune evasion, persistent infection and memory B-cell “immortalization.”^[18]^ In a case-control study recruiting 87 SLE patients meeting the American College of Rheumatology diagnostic criteria and 50 healthy individuals, Chougule D et al found significantly increased levels of EBV antibodies in SLE patients compared with healthy controls (*P* < .05) and a 100% anti-viral capsid antigen (VCA)-IgG positivity rate in SLE patients with kidney involvement.^[19]^ Aygun D et al showed that EBV was associated with altered immune response in SLE, as the rate of EA-IgG positivity was significantly higher in SLE patients (*P* = .005).^[20]^ EBV can express a variety of latent genes in the latent cells, and these genes encode antigens that are highly homologous to certain autoantigens in SLE patients and have the potential to induce autoimmunity, such as the latent genes Epstein-Barr nuclear antigen (EBNA)-1 and EBNA-2. Sundar et al^[21]^ successfully induced anti-Smith (anti-Sm) and anti-dsDNA antibodies in mice using EBNA-1, suggesting that anti-Sm and anti-dsDNA antibodies cross-react with anti-EBNA-1 antibodies in SLE patients. Incaprera et al^[22]^ found that 50% of SLE patients could be induced to produce IgG antibodies against the synthetic peptide fragment of EBNA-2, whereas normal EBV seropositive populations did not produce such antibodies. The distinctive characteristics of SLE pathology is the excessive proliferation and activation of B lymphocytes, resulting in the production of a large number of multiple autoantibodies. The role of EBV in the pathogenesis of SLE may be the massive activation of B lymphocytes, which leads to the development of the disease, and the active virus can further promote the progression of the disease. EBV infection can cause clinical manifestations similar to SLE on the one hand, and may be a predisposing and exacerbating factor for SLE on the other, which are closely related. Therefore, we do not exclude the presence of symmetrical polymorphic erythema of the face, lowering of both tracts, and liver and kidney damage as subclinical manifestations of SLE in children with EBV infection. We recommend long-term follow-up for such children, and further renal biopsy should be performed to identify any tendency of disease progression, such as recurrent vasculitis, persistent hematuria and proteinuria, and autoantibodies turning positive. Therefore, we do not exclude the presence of symmetrical polymorphic erythema of the face, decline in granulocyte and megakaryocyte lineages, and liver and kidney injury as subclinical manifestations of SLE in children with EBV infection. We recommend long-term follow-up for such children, and further renal biopsy should be performed to identify any tendency of disease progression, such as recurrent vasculitis, persistent hematuria and proteinuria, and autoantibodies turning positive.

## 5. Conclusion

We reported a case of chronic EBV infection with facial persistent EM and multi-system performance. The clinical manifestations of EBV infection are complex and varied, and are associated with a variety of immune diseases such as SLE, HSP, and dermatomyositis, so clinical workers need to pay attention to identifying multiple organs and systems injury caused by EBV infection. The prognosis for EBV infection varies, and aggressive antiviral therapy is recommended for EBV-infected patients with clinical symptoms to block the immune process involved.

## Acknowledgments

We would like to thank the patient and her family for providing informed consent for the publication of this case report. This research was supported by the Science and Technology Bureau of Sichuan province (No. 21ZDYF1329).

## Author contributions

Guo Hui conceptualized and supervised the study, Peng Fenfang collected clinical data, conducted the follow-up, wrote the manuscript, which all authors reviewed before submission.

**Conceptualization:** Peng Fenfang, Guo Hui.

**Data curation:** Guo Hui.

**Funding acquisition:** Guo Hui.

**Methodology:** Guo Hui.

**Resources:** Guo Hui.

**Supervision:** Guo Hui.

**Writing – original draft:** Peng Fenfang.

**Writing – review & editing:** Peng Fenfang, Guo Hui.
